# Understanding cavity dynamics near deformable oil drop via numerical simulations^☆^

**DOI:** 10.1016/j.ultsonch.2025.107325

**Published:** 2025-03-24

**Authors:** Deepak K. Pandey, Rupak Kumar, Vivek V. Ranade

**Affiliations:** Multiphase Reactors and Intensification Group, Bernal Institute, University of Limerick, Limerick V94T9PX, Ireland

**Keywords:** Cavity-droplet interaction, VOF, Cavity/ droplet size, Cavity jet velocity, Energy dissipation rate

## Abstract

Cavitation is increasingly being used for producing liquid–liquid emulsions. Cavity collapse generates microscale high-speed jets, which play a crucial role in cavitation-driven emulsification. It is thus essential to investigate the interaction of cavity and droplet to improve the understanding of the cavitation-driven emulsification process. In this study, we have numerically investigated the interaction of a single cavity-droplet pair dispersed in a water medium mimicking the scenario occurring inside a hydrodynamic cavitation-based fluidic device. A direct numerical simulation utilizing the multi-fluid, volume of fluid (VOF) method has been used for simulating different scenarios of cavity droplet interactions. The effect of the droplet-cavity size ratio (β) and the stand-off parameter (γ) on cavity-droplet dynamics have been investigated. The influence of these parameters on cavity jet velocity $\left(U_{\max}\right)$ and energy dissipation rate (ε) was evaluated. Cavity jet velocity ($U_{\max}$) increases at first, then decreases with the stand-off parameter whereas it increases and becomes almost constant for the size ratio. The maximum cavity jet velocity in the present work is obtained for the case β=2.5(γ=0.7) and β=5(γ=1.2). The energy dissipation rate for cavity-oil droplet interaction is of the order $\;10^{8}$ m^2^/s^3^, irrespective of the stand-off parameter and size ratio for a given driving force. The results presented in this work improve the current fundamental understanding of cavity–drop interactions and provide a useful basis for developing cavitation-induced droplet breakage models for predicting droplet size distributions, enabling enhanced applications of cavitation for emulsification in the chemical industries.

## Nomenclature

B: Empirical parameter, MPa
L: cInitial distance between the cavity surface and the oil droplet surface, μm
N: Adiabatic exponent, −
P: Pressure, Pa
R: Radius, μm
*t*: Time, μs
T: Temperature, K
U, *u*: Velocity, m/s

**Greek letters**
α: Volume fraction, −
β: Size ratio, −
ρ: Density, kg/m^3^
μ: Dynamic viscosity, mPa.s
τ: Stress tensor, N/m^2^
*p*: Pressure, Pa
σ: Surface tension, N/m
∇.: Divergence operator, −
∇: Gradient operator, m^-1^
κ: Surface curvature, m^-1^
**γ**: Stand-off parameter, −
*ε*: Energy dissipation rate, m^2^/s^3^
$\overset{¯}{\varepsilon }$: Volume-weighted average energy dissipation rate, m^2^/s^3^
δ: Kronecker delta function
φ: Stand-off parameter when $R_{d}\to \infty$, −

**Subscript**
a: Air
c: Cavity
d: Drop
*i, j*: Phase pairs
o: Oil
*t*: Time
w: Water

**Acronyms**
FVM: Finite Volume Method
CSF: Continuum surface force model
VOF: Volume of Fluid
EoS: Equation of State
BIM: Boundary Integral Method
LBM: Lattice Boltzmann Method
PIMPLE: Pressure-Implicit Method for Pressure-Linked Equations
SIMPLE: Semi-Implicit Method for Pressure-Linked Equations
PISO: Pressure-Implicit with Splitting of Operators
MULES: Multidimensional universal limiter with explicit solution
NCG: Non-condensable gas
PIV: Particle Image Velocimetry

## 1 Introduction

Liquid-liquid emulsions are widely used in various industrial sectors such as healthcare, personal care, home care, food and nutrition, and agrochemicals. Hydrodynamic cavitation presents an attractive alternative for producing liquid–liquid emulsions [1]. Several designs of hydrodynamic cavitation devices are available and have been used for generating emulsions [2]. Droplet breakage models are essential for device scale simulations for accurate prediction of droplet size distributions (DSD). An accurate estimation of energy dissipation rates (ε) in the emulsification devices is needed for such droplet breakage models. Several researchers have investigated the relationship between droplet breakage and the ε in turbulent flow fields [3], [4], [5], [6], [7], [8], as ε plays a crucial role in determining the extent of droplet deformation and fragmentation. Most of the previous works on turbulent breakage models are based on energy dissipation rates predicted using various turbulence models. None of these models are suitable to capture highly localized intense energy dissipation rates realized by the collapse of cavities in hydrodynamic cavitation-based emulsification devices. Previous work of our group [9] attempted to predict droplet size distributions (DSD) generated by hydrodynamic cavitation devices and showed that it is essential to account for localized energy dissipation rates caused by collapsing cavities for realistic simulations of emulsification by hydrodynamic cavitation devices [10]. The localized energy dissipation rates generated by cavity collapse will be influenced by neighbouring oil droplets. It is therefore essential to improve the fundamental understanding of the cavity–droplet interactions for simulating localized energy dissipation rates and shear generated by collapsing cavities in the presence of oil droplets. Such an attempt is made here which will be useful for understanding and developing better computational models for cavitation-induced droplet breakage.

**Fig. 1:**
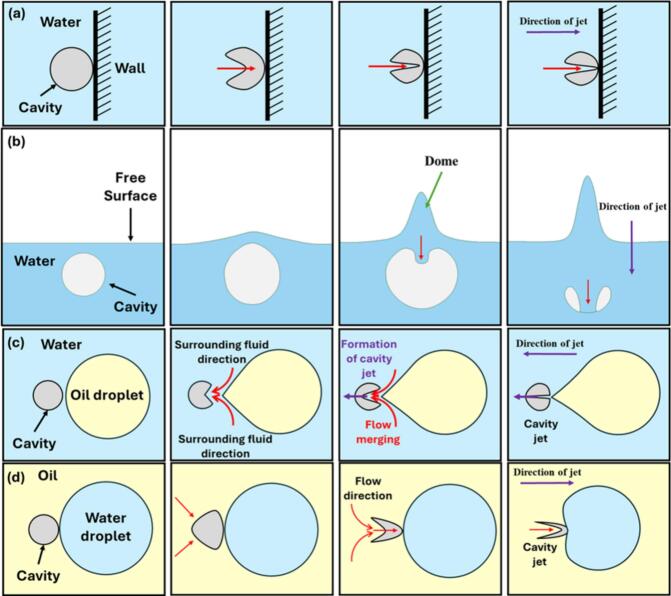
Schematic representation showing directionality of cavity jet in (a) water-cavity-wall system [24]; (b) water-cavity-free surface system [31]; (c) oil-cavity-water drop system [32]; (d) water-cavity-oil drop system [22], [32].

In the past, cavity dynamics was extensively studied by several researchers. These investigations started with an analytical description of a simple model, a spherical cavity in an incompressible, inviscid liquid [11]. This model was extended by including the effect of surface tension and viscosity of the liquid, called the Rayleigh-Plesset model [12]. Even more involved models were formulated considering the compressibility effects, for instance, the Gilmore model [13] and the Keller-Miksis model [14]. For coping with aspherical cavity dynamics, the boundary integral method (BIM) was developed by Blake and Gibson [15]. This method allows the simulation of aspherical cavity dynamics in incompressible and weakly compressible liquids. It can simulate the jet formation upon aspherical cavity collapse [16], [17]. The impact of the collapsing cavity on the object (wall) present in its vicinity has been accounted for by Pandit et al. [18] in terms of impact pressure. Moreover, the radial velocity generated by the oscillation of the cavity was used to obtain the turbulent energy dissipation rate. These models mostly consider spherical collapse and do not include the complexity that arises from the multiphase interface, such as free surface, solid objects, and fluids of different physical properties. The presence of an interface in the proximity of a cavity, not only influences the velocity but also the directionality of the high-velocity jet [11], [12], [13], [14], [15]. The influence of nearby interface on cavity dynamics and resulting high-velocity jet is shown schematically in Fig. 1.

In the case of solid objects such as rigid walls [24], [25] or spheres [26], [27], it has been reported that the cavity collapses asymmetrically and produces a micro-jet towards the rigid wall as shown in Fig. 1(a) [21], [25], [28]. The stand-off parameter between the wall and the cavity dictates the strength of the micro-jet [25]. The maximum velocity of the micro-jet has been reported for an optimum stand-off parameter [27], [28], [29], [30]. Brujan et. al [19] studied cavity dynamics by generating a cavity between two equidistant perpendicular rigid walls. It was observed that the jet formed upon cavity collapse made an angle of 45^0^ with both walls [19]. In the case of cavity dynamics near free surfaces [28], [29], the cavity elongates slightly along the axis, while a free surface hump is formed due to the push (expansion) from the cavity, as shown in Fig. 1(b). Before reaching the maximum volume, the top part of the cavity margin (margin refers to the region of water between the cavity and the free surface) expands into the dome and turns to contract. The cavity collapses with a flat top and leads to a thin liquid jet. Meanwhile, the dome keeps rising and contracts in width, leading to the formation of the water spike (refer to Fig. 1(b)) [28]. Furthermore, Ohl et. al [20] investigated the influence of a thin oil layer between the cavity and the wall. It was found that the presence of the oil layer between the cavity and wall did not influence the direction of the cavity jet.

Some studies also have been conducted on cavity-droplet interactions. Orthaber et al. [22] studied the interaction between the cavity and the oil droplet $\left(R_{d}\;\;∼\;\infty \right)$ inside the water domain. It was observed that when the cavity was generated inside the water, the cavity jet moved away from the oil–water interface, that is towards the water. The cavity jet direction was also found to be towards the water when the cavity was generated inside the oil [23], [30], [33]. Raman et al. [34] numerically and experimentally studied the interaction between the cavity and water droplet in the continuous medium of silicon oil and evaluated the effect of viscosity. During the cavity collapse in a higher viscous medium, the droplet elongates towards the cavity, which acts as a flow sink pulling the droplet. The droplet jets into the cavity and forms satellite water droplets. In the case of lower viscous oil, the droplet encapsulates the collapsing cavity as it jets inside and undergoes multiple cycles of expansion and collapse. Cavity collapses creating tiny oil droplets inside the water droplet. Yamamoto and Komarov [32] studied the cavity collapse in the vicinity of the oil droplet inside water where the cavity was generated by acoustic cavitation. The influence of the distance between the cavity and droplet on collapse dynamics was investigated. They found that the cavity jet formed is directed towards the water, that is away from the oil droplet (Fig. 1(c)) [32]. The cavity jet velocity depends on the distance between the initial cavity and the droplet distance. In addition, the direction of the cavity jet was also influenced by the physical properties of the droplet (Fig. 1(d)). Yin et al. [35] investigated the interaction between the cavity near the wall with a hemispherical air cavity between the cavity and the wall. The direction of the cavity changes when the size of the air cavity increases, that is cavity jet is away from the wall. When the air cavity size was small, the cavity jet was towards the wall. Moreover, when the stand-off distance is less, the cavity jet is away from the wall. As the stand-off distance increases, the cavity jet direction formation may not occur.

It is evident from the above discussion that most of the studies involving cavity-droplet interaction have focused on directionality, the effect of the stand-off parameter, and the effect of physical properties. However, the influence of stand-off parameters and size ratios on droplet-cavity interaction including the cavity jet directionality and resulting energy dissipation rates has not been investigated for hydrodynamic cavitation-based fluidic devices. These aspects are important for gaining a better understanding of the cavity-induced emulsification process. In this work, the cavity dynamics in the vicinity of a deformable oil droplet have been studied using direct numerical simulations (DNS). The influence of the different stand-off parameters and size ratios has been investigated. The generated energy dissipation rates due to the formation of the cavity jet have been calculated. The numerical methodology, grid independence test, validation and cavity dynamics are discussed in 2, 2.1 respectively. The present study will provide a useful basis for developing appropriate drop breakage models for hydrodynamic cavitation-based fluidic devices.

## 2 Computational model

**Fig. 2:**
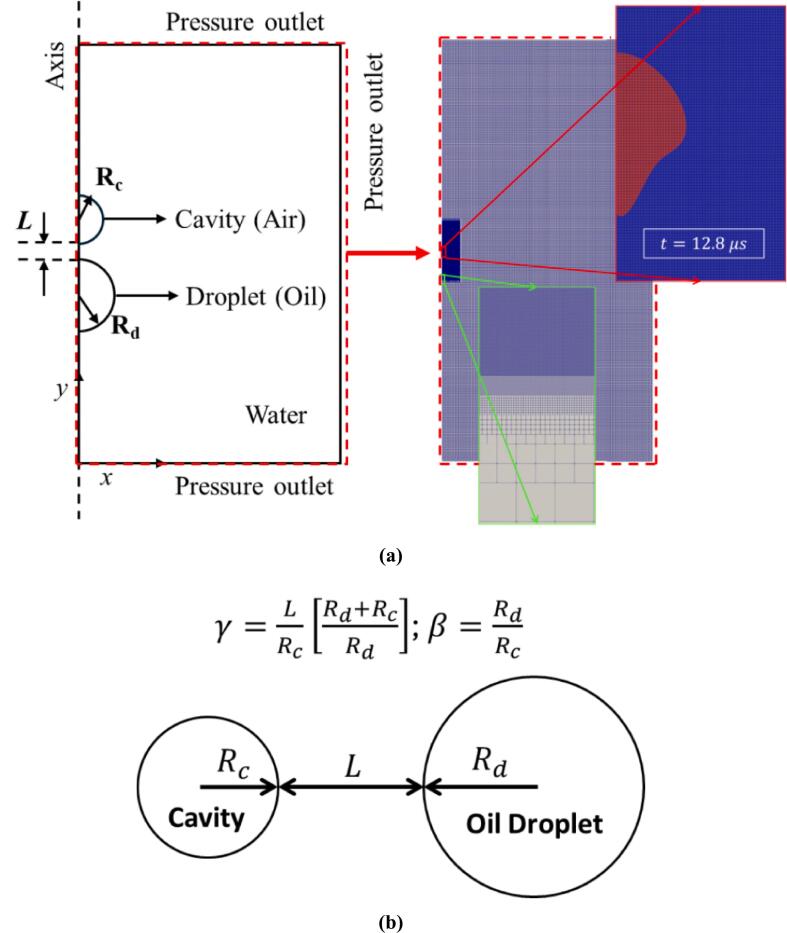
(a) Computational domain and grid distribution; (b) Stand-off parameter (γ) and size ratio (β) used in the present study.

The interaction between a cavity (air) and a droplet (oil) dispersed in a continuous liquid (water) is investigated in this work. The schematic of the problem is shown in Fig. 2(a). The standard model for cavity dynamics primarily focuses on inertial forces, disregarding mass and heat diffusion across the cavity wall as well as phase change effects [20], [36]. This model applies to cavities in water at normal ambient temperatures. In this context, the dynamics of the cavity are driven mainly by inertia and compressibility effects, with heat diffusion being negligible. Experimental evidence by Vogel et al. [37] also supports this, showing that over 80 % of the cavity energy is released as a shock wave during a strong cavity collapse. Gas diffusion through the cavity wall is also negligible because of its time scale ($\;10^{-1}-10^{0}$ s) is much longer than the cavity oscillations considered in this study ($\;10^{-6}$ s). Thus, thermodynamic effects have minimal impact on the overall dynamics of the cavity. The cavity filled with non-condensable gas (NCG) [38] can behave differently from the cavity filled with water vapour. This behaviour change is attributed to phase change phenomena. The cavity filled with water vapour decreases the cavity pressure over time and, hence, the cavity size due to the condensation effect [34]. To account for this behaviour, a one-time correction to cavity pressure was included, which reduces the cavity pressure by a factor of 0.35, once the cavity reaches its maximum size during the first cycle of oscillation [34]. A similar internal pressure treatment was used by Fan et al. [39] where the radius of the initiated cavity was reduced by a factor when it reached its maximum size. This correction accounts for the partial condensation of cavity contents not considered in the non-condensable gas assumption to match the experimental results in the second oscillation. The use of NCG simplifies the model. Isothermal conditions have been assumed because heat conduction [36], [37] does not significantly affect the fast cavity dynamics. The analysis includes the liquid’s viscosity and the interfacial tension as these factors significantly impact the dynamics of the cavity and the interaction between cavity droplets [36].

The physical phenomena of cavity droplet interaction are symmetrical about the central axis; hence, an axisymmetric study has been undertaken in the present work. All the fluids are taken as Newtonian. The continuous medium (water) and the oil droplet are (nonlinearly) treated as compressible and the density of each phase is represented by Tait’s equation of state [23], [36], [40]. The oscillations of a cavity are initiated by specifying an elevated pressure which is significantly higher than the other two phases which are at atmospheric pressure conditions. The model equations, boundary conditions and solution strategies used for the simulations are discussed in the following section.

### 2.1 Governing equations

A single set of governing equations (mass, momentum, and energy conservation) is solved to calculate the flow fields. The equations are given as follows:

(1) $$\frac{\partial \rho }{\partial t}+\nabla \cdot \left(\rho u\right)=S_{c}$$

(2) $$\frac{\partial \left(\rho u\right)}{\partial t}+\nabla \cdot \left(\rho uu\right)=\;-\nabla p+\nabla ∙\left(\mu \nabla u\right)+\rho g+F_{s}+S_{m}$$

(3) $$\frac{\partial \left(\rho C_{p}T\right)}{\partial t}+\nabla \cdot \left(\rho C_{p}uT\right)=\;\nabla ∙\left(k\nabla T\right)-p\nabla ∙u+S_{e}$$

where u, p, T, ρ, μ, $C_{p}$, k, g, $F_{s}$, $S_{c}$, $S_{m}$, and $S_{e}$ are velocity, pressure, temperature, mixture density, mixture viscosity, mixture specific heat, mixture conductivity, gravity of acceleration, surface tension force, and source terms for mass, momentum, and energy, respectively. The presence of multiple phases is modelled using a multi-fluid algebraic volume of fluid (VOF) approach. The VOF method has been utilised to capture interfaces between different phase pairs, as it can rigorously conserve mass and handle complex interfaces [41]. This method has been effectively applied in previous research by Raman et al. [34] to investigate cavity-droplet interactions and is utilized here to analyse the cavity dynamics in the vicinity of oil droplet. The choice of FVM with VOF over the Boundary Integral Method (BIM) and Lattice Boltzmann Method (LBM) is due to its capability to capture sharp interfaces, handling large deformations and topology changes [33], [37], [41], making it suitable for simulating detailed cavity-droplet interactions with irregular shapes. Additionally, FVM with VOF can seamlessly integrate viscous effects and surface tension forces, which are essential for accurately modelling the dynamics of these interactions [33], [41]. In the VOF method, scalar transport equations (Equation 4) are solved using a multidimensional universal limiter with explicit solution (MULES) to calculate volume fraction distribution (Equation 5) in each cell [35], [36].

(4) $$\frac{\partial \alpha_{i}}{\partial t}+\nabla \cdot \left(u\alpha_{i}\right)=0,\;\;i=1,\;2,\;3$$

(5) $$\alpha =\frac{V_{i}}{V}$$

The sum of the volume fractions of all the phases in a cell is always equal to one (Equation 6).

(6) $$\sum \alpha_{i}=1$$

The mixture properties such as density, viscosity, heat capacity, and conductivity are determined using Equation 7.

(7) $$b\;=\sum \alpha_{i}b_{i}\;,\;\;\;\;i=1,2,3,\;\;\;b\in \left(\rho ,\;\mu ,\;C_{p},\;k\right)$$

To account for the surface tension force at the interfaces, a source term ($F_{s}$) is included in the momentum equation (Equation 2). It is based on the continuum surface force model (CSF) given by Brackbill et al. [42]. The surface tension force ($F_{s}$) is given as:

(8) $$F_{s}=\sum_{i}\sum_{j\neq i}\sigma_{\mathrm{ij}}\kappa_{\mathrm{ij}}\delta_{\mathrm{ij}}$$

where, $\sigma_{\mathrm{ij}}$, $\kappa_{\mathrm{ij}}$, and $\delta_{\mathrm{ij}}$ are surface tension coefficient, curvature, and Kronecker delta function respectively. Subscripts i, j denote different phase pairs. The curvature ($\kappa_{\mathrm{ij}}$) is calculated using:

(9) $$\kappa_{\mathrm{ij}}=-\nabla ∙\left(\frac{\alpha_{j}\nabla \alpha_{i}-\alpha_{i}\nabla \alpha_{j}}{\left|\alpha_{j}\nabla \alpha_{i}-\alpha_{i}\nabla \alpha_{j}\right|}\right)$$

and the Kronecker delta function ($\delta_{\mathrm{ij}}$) is given as:

(10) $$\delta_{\mathrm{ij}}=\alpha_{j}\nabla \alpha_{i}-\alpha_{i}\nabla \alpha_{j}$$

Here, $\delta_{\mathrm{ij}}$ ensures that the volumetric surface tension force acts only at the interface.

Tait equation of state (Equation 11) is used to account for the compressibility in the liquid and gaseous phases, as it models fluid density over a range of pressures (0.1 MPa–50 MPa), that is, high pressure during cavity collapse and low-pressure during cavity growth [40], [43]. It also ensures the effective modelling of the nonlinear compressibility of liquid and vapour phases and provides a direct link between EoS and isentropic bulk modulus [44].

(11) $$p=\left(p_{0}+B\right){\left(\frac{\rho }{\rho_{0}}\right)}^{N}-B$$

Here, $p_{0}$, $\rho_{0}$, N, and B are reference pressure, reference density, adiabatic exponent and empirical parameter respectively. For B=0, it reduces to the ideal gas equation. Properties for different fluids are taken from the study of Raman et al. [34] and given in Table 1. Here we use the adiabatic approximation to model the gas pressure inside the cavity since the dynamics of the cavity are driven mainly by inertia and compressibility effects, with heat diffusion being negligible [37].

**Table 1:** Parameters and their values utilized in Equation 11[34].

**Parameter**	**Air**	**Water**	**Oil**
Reference density, ($\rho_{0},$ kg/m^3^)	0.12	998.2	960
Reference pressure ($p_{0}$, Pa)	10,320	101,325	101,325
Adiabatic exponent (N)	1.33	7.15	6.4
Empirical parameter (B, MPa)	0	404.6	150

### 2.2 Computational domain and boundary conditions

As mentioned earlier, an axisymmetric computational domain is considered. The computational domain represents a part of a large cylindrical domain filled with a continuous phase (water), where a cavity (air) of radius, $R_{c}$ and a droplet (oil) of radius, $R_{d}$ are placed in proximity with a centre-to-centre distance $\left(L+R_{c}+R_{d}\right)$ (Fig. 2(b)). The size of the domain is taken as 100 times the maximum radius of the cavity to minimise the influence of boundary conditions on the cavity droplet interaction. Pressure outlet and wave-transmissive boundary conditions are used at the outlets to mimic a larger fluid domain. Initially, the entire domain is at the atmospheric condition except the cavity. The cavity was given the initial condition similar to the cavity which is generated inside a hydrodynamic cavitation-based fluidic device. To find the initial condition of the cavity generated via hydrodynamic cavitation, Yin et al. [45] work has been referred to. Yin et al. [45] conducted experiments and numerical simulations using the snapping claw experiment (which is based on pistol shrimp and mimics cavities generated via hydrodynamic cavitation) and obtained a pressure of $\;10^{7}$ Pa which was also validated by analytical calculations. Moreover, Koukouvinis et al. [46] also stated that the maximum pressure generated during the cavitation by a pistol shrimp is 80-100 bar ($8x10^{6}$ Pa– $10^{7}$ Pa). Based on these studies, we have specified an overpressure of $\;10^{7}$ Pa in our study.

The size ratio (β) and stand-off parameter (γ) are defined as:

(12) $$\beta =\frac{R_{d}}{R_{c}}$$

(13) $$\gamma =\varphi \left(1+\frac{1}{\beta }\right)$$

Where, $R_{c}$ is the initial radius of the cavity (fixed at 10 μm), $R_{d}$ is the initial radius of the oil droplet, $\varphi =L/R_{c}$, L is the distance between the initial cavity surface to the oil droplet surface as shown in Fig. 2(b). The definition of γ in the present work is different from that of Orthaber et. al [23]. Orthaber et. al [23] defined γ as the ratio of the initial cavity centre to liquid boundary distance by the maximum radius $\left(R_{\max}\right)$ of the cavity achieved in the absence of interference. Orthaber et. al [23] estimated the initial radius of the cavity as $\frac{R_{\max}}{6.25}$. This relationship is used for estimating the initial radius of the cavity in the present work for the comparison of our simulated results with the results of Orthaber et. al [23]. In the case of a planar boundary $\left(R_{d}=\infty \right)$, the relationship between γ of the present work and Orthaber et. al [23] work is:

(14) $$\gamma =6.25\;\gamma_{\mathrm{Orthaber}}-1$$

Thus γ=1.4 (base case in the present work) is similar to $\gamma_{\mathrm{Orthaber}}=0.384$.

The motivation behind this work is to gain insight into the interaction of a single cavity with an oil droplet dispersed in a water medium. Such a situation occurs when hydrodynamic cavitation devices such as vortex diode, venturi and orifice are used for emulsification [1], [2]. The typical lifetime of cavity collapse, liquid velocities in the cavitating region, initial cavity size and typical droplet sizes of oil in water emulsions are estimated based on the previous works [1], [2], [7], [10], [18] and are used for selecting appropriate ranges of the considered parameters γ and β. Based on these estimates, the stand-off parameter (γ) was varied in the range of 0 - 4 and the (β) was varied in the range of 0.3-10. Moreover, the range of Reynolds number (Re), Weber number (We) and Ohnesorge number (Oh) used in the present study has been included in Table 2 for different γ and β. The density selected for the calculation of these dimensionless numbers belongs to the continuous fluid (water in the present work).

**Table 2:** Re, We, Oh for the range of γ and β

γ	β	$Re(10^{3})$	$We(10^{3})$	$Oh(10^{-2})$
∼ 0.00	2.50	3.81	14.3	3.13
0.28	2.50	5.07	25.2	3.13
0.49	2.50	4.88	23.4	3.13
0.70	2.50	5.66	31.4	3.13
1.40	2.50	5.38	3.55	1.11
2.00	2.50	4.73	21.9	3.13
2.80	2.50	4.27	17.9	3.13
4.27	2.50	4.27	17.9	3.13
4.20	0.31	0.51	2.09	8.85
2.60	0.63	1.09	2.31	4.43
1.67	1.50	2.78	2.62	1.84
1.40	2.50	5.38	3.55	1.11
1.20	5.00	11.9	4.34	0.553
1.10	10.00	23.8	4.34	0.277

### 2.3 Numerical solution and grid independence

Numerical simulations are performed using a finite volume-based open-source computational fluid dynamic software OpenFOAM. The solver compressibleMultiphaseInterFoam is utilised in the present study, which has been successfully applied by different researchers to solve the cavity droplet interaction problem [32], [34]. The governing equations are discretised using the finite volume method (FVM). PIMPLE algorithm (a combination of PISO and SIMPLE) has been utilised to solve the discretised governing equations. At first, the density of each phase is calculated using Equation 11. Volume fraction equations are then solved (Equations 4 and 5). Volume fractions are then used to determine the mixture properties (Equation 7) and surface tension forces (Equations 8, 9 and 10). Mixture properties are used in Equations 1, 2 and 3 to determine the flow fields in the domain. Steps are repeated until the final solution time has been reached. An adaptive time stepping has been utilised based on the Courant number, which is set as 0.05. The simulation details required to set the case in OpenFOAM are included in the Supplementary Information along with the steps for post-processing the result.

**Fig. 3:**
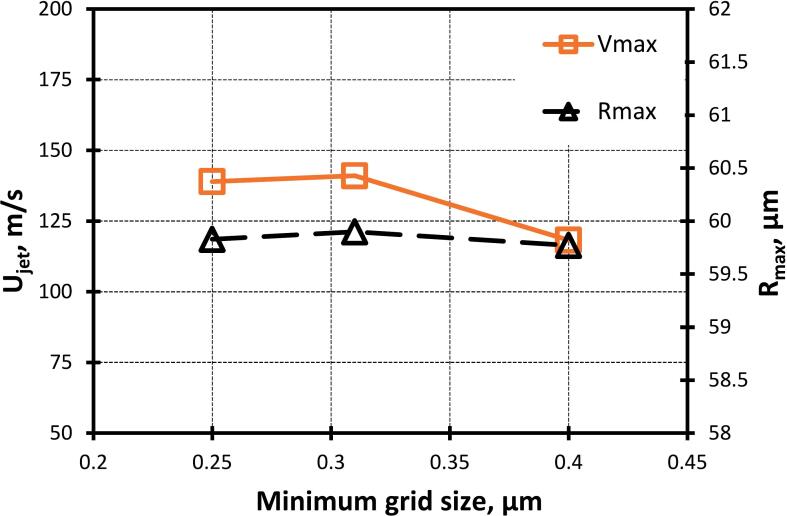
Grid-independence study.

A non-uniform structured Cartesian grid having an aspect ratio of one was used to discretize the simulation domain. It offers a consistent framework for interface capturing, reducing numerical diffusion, and ensuring a precise and sharp interface representation. To enhance the computational efficiency, only the grids around the cavity where the maximum deformation occurs are refined (Fig. 2(a)). Three different grid sizes were considered, that is 0.25 µm, 0.31 µm and 0.4 µm. The influence of grid size on maximum velocity and radius is shown in Fig. 3. Moreover, the Richardson extrapolation method [47] (Table 3) is used with the maximum velocity obtained at t=13 μs as its parameter. The $U_{\max}$ is used because the effect of size ratio (β) and stand-off parameter (γ) on cavity jet velocity has been investigated. Moreover, the cavity jet velocity gradient is used in the evaluation of the energy dissipation rate (ε).

**Table 3:** Minimum grid size and corresponding maximum velocity.

Refinement	Refinement Ratio (r)	Grid size (μm)	Maximum velocity	m/s
h1	1	0.25	V1	139
h2	r21=h2h1=1.24	0.31	V2	141
h3	r32=h3h2=1.29	0.40	V3	118

Using the Richardson Extrapolation formula [47], the value of velocity at mesh size approaches zero ($V_{h\to 0}$) was estimated as:

(15) $$V_{h\to 0}=\;V1+\left(\frac{V1-V2}{{r_{21}}^{p}-1}\right)\;\;\;∼\;135.28\;m/s$$

Where, V1, V2 are the maximum velocities of the cavity jet obtained on grid 1 and grid 2, $r_{21}$ is the grid refinement ratio of grid 2 to grid 1 and p is the order of accuracy, which is 2 for the present case. It can therefore be seen that the absolute percentage error associated with the mesh used in the present work is about 4 %. Based on these results, a minimum grid size of 0.31 µm was chosen for all the subsequent simulations to adequately resolve the cavity dynamics.

## 3 Results and discussions

### 3.1 Verification and validation

**Fig. 4:**
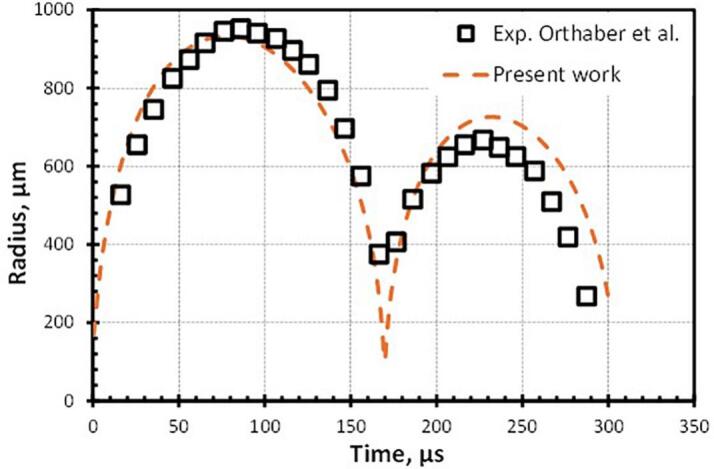
Comparison of the present numerical study with the experimental result of Orthaber et al. [23].

**Fig. 5:**
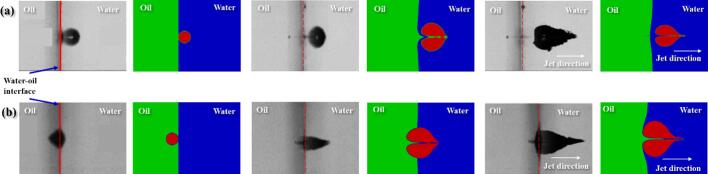
Comparison of cavity jet directionality (a) cavity generated in water; (b) cavity generated in oil (Left: Orthaber et al. [22] and Right: Present work).

The present simulation methodology is first validated by comparing the simulated results with some of the published results of cavity dynamics. It is worth mentioning here that the validation of the numerical simulation has been performed for two different cases. At first, the temporal evolution of a cavity inside a liquid pool is compared with the experimental results of Orthaber et al. [23] in Fig. 4. The quantitative comparison of cavity oscillation is a good match ($R^{2}=0.83$). The temporal discrepancy (Fig. 4) and morphological difference (Fig. 5) in the experimental and simulation results is because simulation does not consider the actual process of cavity formation [23] such as dielectric breakdown, plasma formation, shockwave emission, sub-ambient condition due to fluid flow etc.

In the second case, the numerical model was qualitatively validated against the experimental results of Orthaber et al. [15], as shown in Fig. 5, to understand whether the computational model correctly determines the jet direction in different scenarios. This study was chosen for qualitative validation because the interface between the water and oil is completely flat and simple to simulate numerically. However, even if the interface has a curvature, we believe the numerical model would still agree with the result.

In the case of Orthaber et al. [22], cavity jet directionality has been compared for two cases (a) cavity generated in the water and (b) cavity generated in oil. During the first expansion cycle of the cavity, the pressure waves travel in all directions. However, the propagation speed of the pressure wave is different for the water and the oil medium. The speed of the pressure wave in the oil medium is slower as compared to the water medium. When the cavity is generated inside water, this causes the development of a region of high pressure between the cavity and the oil droplet, and a region of low pressure on the other side of the cavity. This pressure gradient facilitates the formation of a cavity jet away from the oil. The cavity jet creates a suction effect and pulls the surrounding fluid in its immediate vicinity. The surrounding fluid present in the immediate vicinity of the cavity creates a flow-merging effect (refer to Fig. 1(c)). This flow-merging effect facilitates the formation of high-speed cavity jets as shown in Fig. 5(a). When the cavity is inside the oil, a region of high pressure is generated on the cavity side which is away from the water and a low-pressure region on the cavity side which is towards the water. This pressure gradient causes the cavity jet direction towards the oil–water interface. In this case, the cavity is very close to the interface (oil–water) because of which the cavity gets pulled through the interface (as shown in Fig. 5(b)) because of the pressure gradient [22]. In both cases, the cavity jet direction is always towards the water as discussed previously. The present simulations thus correctly captures the directionality of the cavity jet direction (as shown in Fig. 5) for both the cases (when cavity is generated in oil and when cavity is generated in water).

### 3.2 Cavity droplet interaction

**Fig. 6:**
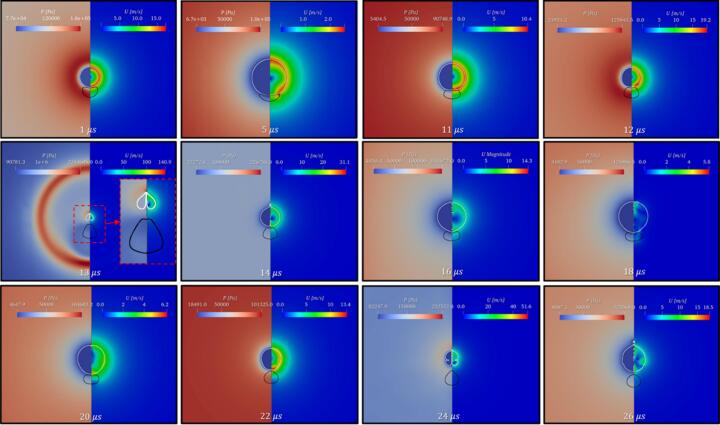
Pressure (Left) and velocity (Right) contours for β=2.5 at γ=1.4.

**Fig. 7:**
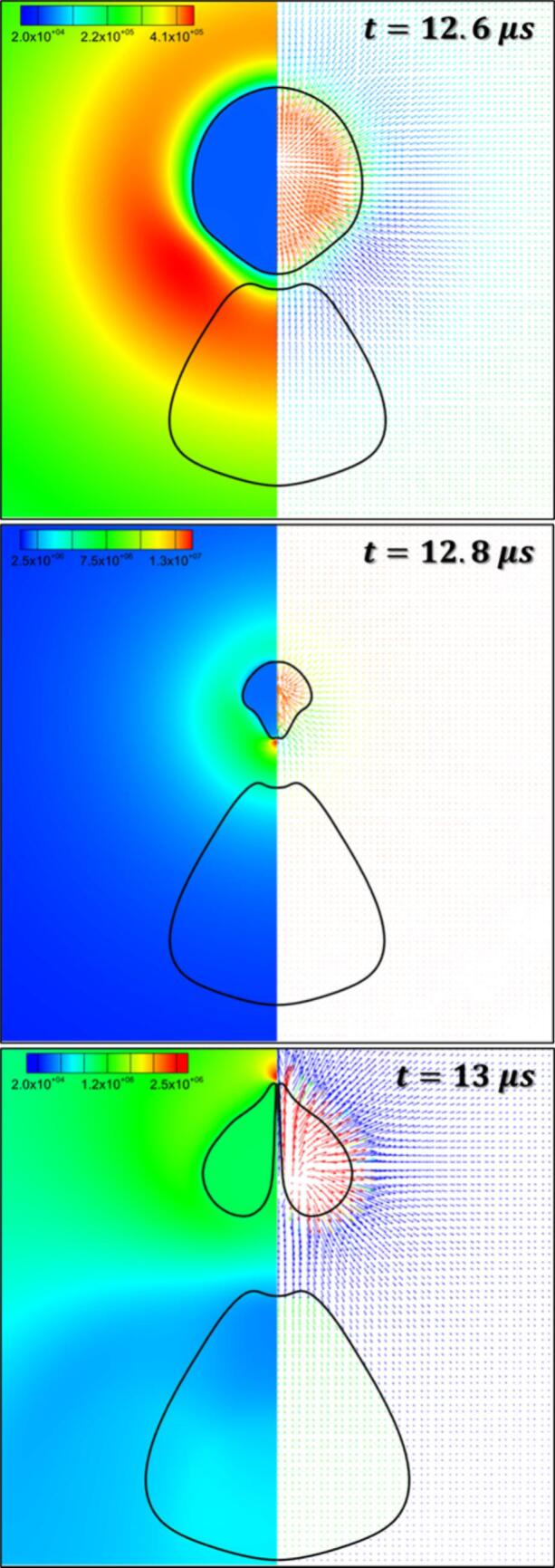
Pressure (Left) contour and velocity (Right) vector for β=2.5atγ=1.4 for t=12.6 μs, 12.8 μs and 13 μs.

In this section, the cavity-oil droplet interaction is carried out for the case where the cavity’s radius, $R_{c}=10$ μm, oil droplet’s radius, $R_{d}=25$ μm, stand-off parameter, γ=1.4, initial pressure inside the cavity as $P_{b0}=10^{7}$ Pa, and atmospheric conditions everywhere else, have been discussed. The pressure difference between the cavity and the surroundings is the driving force behind the cavity oscillations. The simulated results are shown in Fig. 6. As time progresses, the cavity grows (first expansion), pushing the continuous liquid (water) away, and making direct contact with the droplet which is present in its vicinity as shown in Fig. 6 at t=1 μs. This expansion of the cavity subsequently deforms the droplet into a C-shaped structure (Fig. 6, t=5 μs). This expansion process continues till the cavity reaches its maximum radius ($R_{\max}$). Thereafter, the internal cavity pressure ($P_{b}$) reaches a minimum (t∼6.6 μs). Upon attaining the minimum pressure, the process of the first cavity collapse begins. Since the collapse process is very fast, it leads to the generation of pressure waves inside the continuous liquid. The pressure wave travels relatively slower in the oil medium than in the water. This facilitates the development of a region of relatively high pressure adjacent to the oil droplet refer to Fig. 7 at t=12.6 μs. As the cavity collapse progresses, a region of high pressure is observed between the cavity and the oil droplet as shown in Fig. 7 at t=12.8 μs and a region of low pressure is on the opposite side of the cavity. The pressure difference across the cavity facilitates the formation of the cavity jet during the first collapse cycle. This cavity jet creates a suction effect and pulls the surrounding fluid present in its immediate vicinity. Because of this pressure difference across the cavity, a flow merging effect is created as shown in Fig. 1(c). This flow-merging effect facilitates the formation of high-speed water jets (>140 m/s) known as a cavity jet as shown in Fig. 6, Fig. 7 at t=13 μs and is also responsible for the deformation of the droplet. Thereafter, the second cycle of oscillation begins which is shown in Fig. 6 at t=14 μs. During the second expansion, the cavity jet vanishes and the cavity merges together (refer to Fig. 6 at t=16 μs). These oscillations will continue till the cavity loses its driving force.

**Fig. 8:**
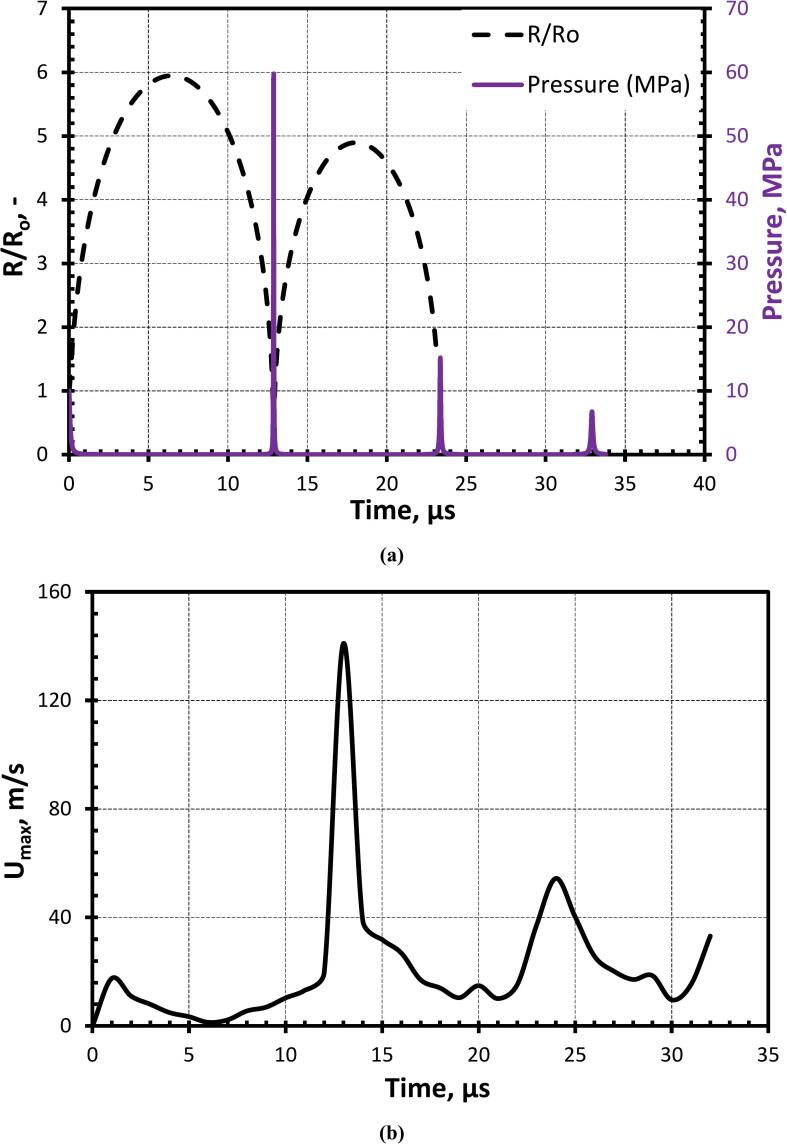
Temporal variation of (a) radius (black colour) and pressure (purple colour) of the cavity; (b) cavity jet velocity for β=2.5 at γ=1.4

Fig. 8(a) quantitively shows the expansion-compression cycle of the cavity for the case, β=2.5 at γ=1.4. The cavity expands up to 6 times its initial radius when an overpressure of $10^{7}$ Pa was applied to the cavity. As discussed previously, a pressure gradient (∼60 MPa shown in Fig. 8(a)) develops across the cavity, leading to the formation of the cavity jet with a velocity ($U_{\max}$) of ∼140.9 m/s (as shown in Fig. 8(b)). This is in good agreement (within 0.1 %) with the maximum velocity obtained by using Equation 20 of Pandit et al. [18]. Considering the equivalent value of stand-off parameter ($\gamma_{\mathrm{Orthaber}}=0.384$ which is equivalent to γ=1.4 as used in the present work) and using the Equation 1 of Dular et al. [48], the cavity jet velocity is 131.73 m/s. It can thus be seen that for equivalent values of stand-off parameters, the cavity jet velocity obtained in the present work agrees with that of Dular et al. [48] within 7 %.

### 3.3 Effect of stand-off parameter (γ)

**Fig. 9:**
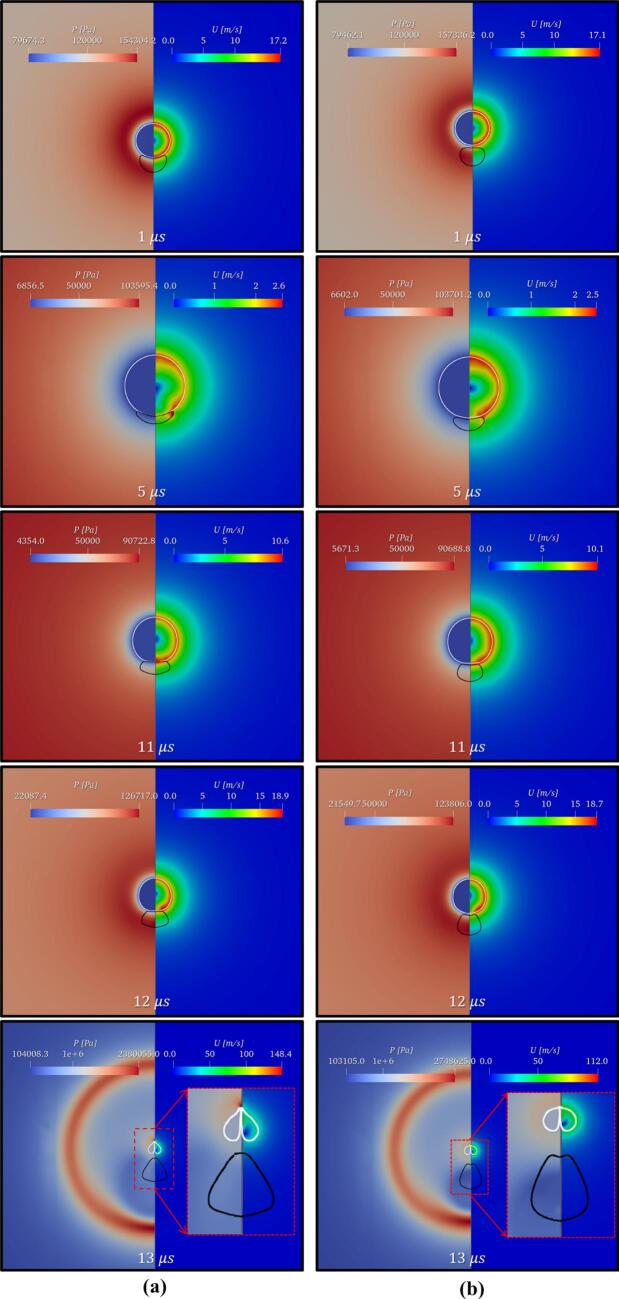
Pressure (Left) and velocity (Right) contour for different stand-off parameters (γ) (a) γ=0.70; (b) γ=2.8 atβ=2.5

**Fig. 10:**
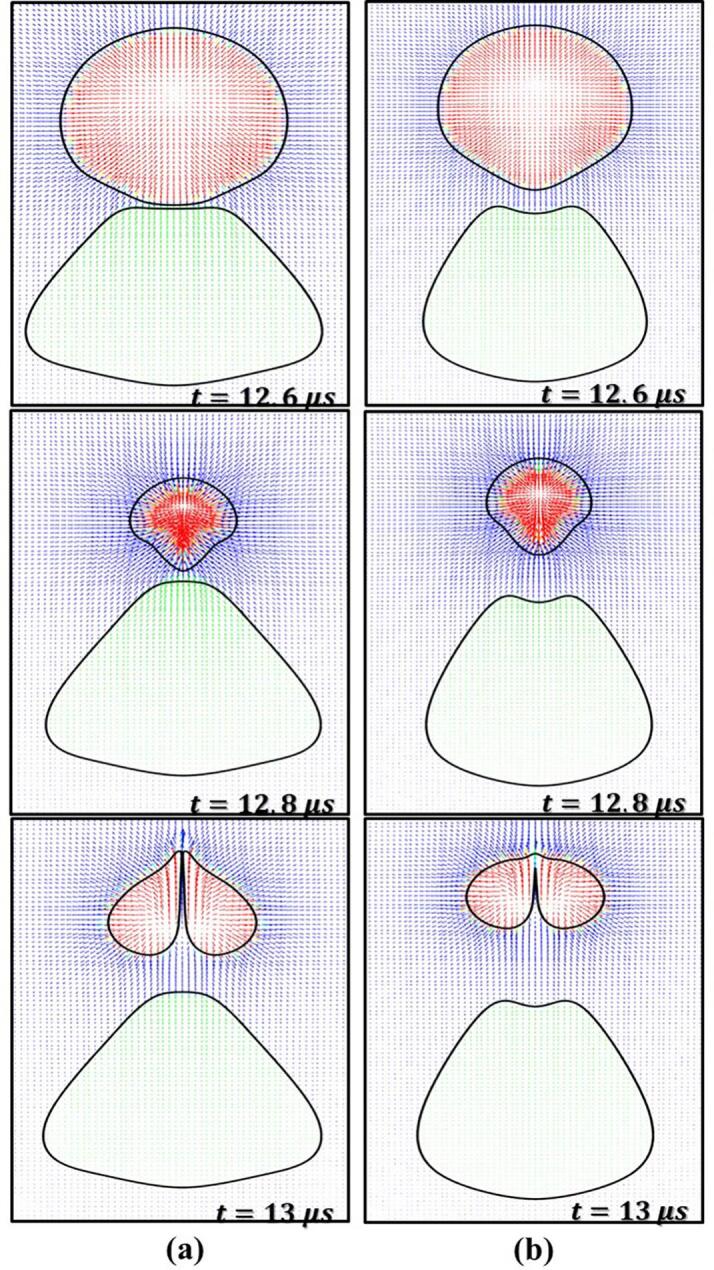
Velocity vector for γ=0.7 and γ=2.8atβ=2.5 for t=12.6 μs, 12.8 μs and 13 μs.

In this section, the effect of the stand-off parameter (γ) on cavity droplet interaction has been investigated. The size ratio (β) is kept constant at 2.5, only φ is varied by varying L as $R_{c}=10$ μm. The cavity dynamics during the first expansion of the cavity is similar to the one discussed in Section 3.2. The change in cavity dynamics occurs during the first collapse. Therefore, only the phenomenon occurring during the cavity collapse phase is discussed in this section. The effect of γ influences the pressure and velocity field which in turn will influence the cavity and the droplet interaction characteristics. A wide range of γ (refer to Table 2) has been chosen for this study. Fig. 9 shows the cavity and droplet interaction and corresponding pressure and velocity fields for γ=0.7 and 2.8 at β=2.5. Fig. 10 shows the velocity vector for the same case for t=12.6,12.8 and 13 µs, respectively.

During the cavity collapse process, the oil droplet provides resistance to the flow of the surrounding liquid (water) which leads to the development of a region of relatively high pressure adjacent to the oil droplet and a region of low pressure is already present inside the cavity as shown in Fig. 9 at t=12 μs. This region of high pressure is not only responsible for the distortion of the cavity but also for the elongation of the oil droplet (Fig. 10 at t=12.6 μs). As the cavity collapses proceeds further, the cavity gets more distorted and the oil droplet elongates further. Moreover, a pressure difference is developed across the cavity, that is a high-pressure region between the cavity and droplet and a region of low pressure on the other side of the cavity (similar to Fig. 7 at t=12.8 μs). The pressure gradient across the cavity facilitates the formation of the cavity jet. During the formation of the cavity jet, the momentum of the surrounding liquid due to the flow merging effect is large which causes the droplet to deform into a conical-like structure as shown in Fig. 10 at t=13 μs. Moreover, the higher momentum of the surrounding liquid (water) during the flow-merging also leads to a higher cavity jet velocity (∼148 m/s) as shown in Fig. 9(a) at t=13 μs.

As the γ(=2.8) increases the distance between the cavity and droplet also increases which allows the droplet to elongate due to the movement of the water towards the cavity during its collapse as shown in Fig. 9(b) at t=12 μs and 11(b) at t=12.6 μs. Thereafter, a pressure gradient develops across the cavity as discussed in the previous paragraph. The momentum of water present in the vicinity of the cavity during the flow merging is mostly utilized for the deformation (elongation) of the oil droplet (Fig. 10(b) at t=12.8 μs). Thus, a small portion momentum of the water due to flow merging contributes to the velocity of the cavity jet (Fig. 9(b) at t=13 μs) which is why the magnitude of the cavity jet velocity is reduced (∼112 m/s).

**Fig. 11:**
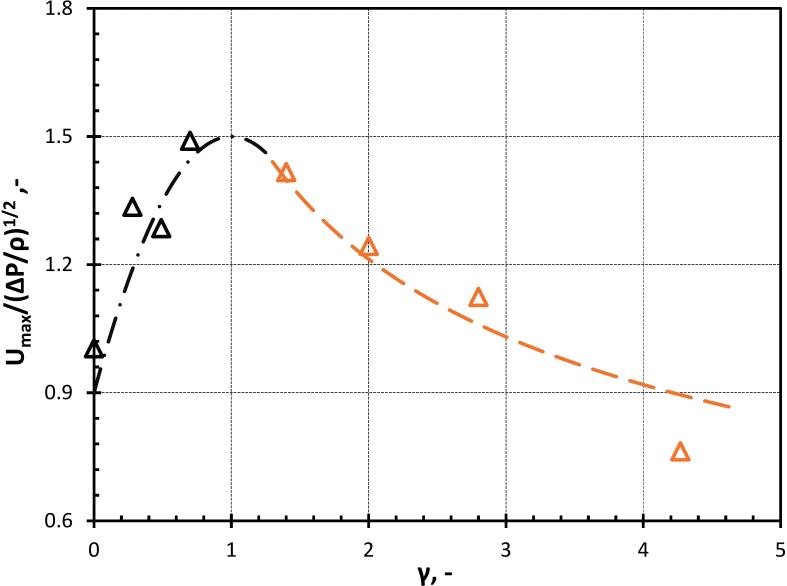
Normalized cavity jet velocity for different stand-off parameters (γ) (black line represents Equation 16 for γ<1.25 and the orange line represents Equation 16 for γ≥1.25).

Fig. 11 shows the variation in the normalised cavity jet velocity with γ till ∼1. This variation of gamma has been obtained by varying φ (refer to Equation 13). As γ increases, the normalised cavity jet velocity also increases because a large portion of momentum due to flow merging assists the cavity jet velocity instead of deforming the oil droplet as shown in Fig. 11. For γ≥1, the distance between cavity and droplet increases which provides more space for the droplet to elongate and the influence of the droplet on flow merging also reduces. The normalised cavity jet velocity starts decreasing as a major chunk of the water momentum due to flow merging is utilized in the elongation of the oil droplet (as discussed previously). For a very high value of γ(>>4), the influence of the droplet will no longer influence the cavity dynamics. Thereafter, the cavity jet formation may not occur. A correlation has been proposed relating the variation of the normalized cavity jet velocity with γ as:

(16) $$\frac{U_{\max}}{{\left(\Delta P/\rho \right)}^{\frac{1}{2}}}=\left\{\begin{array}{c} 1.5\left[1-0.4{\left(\gamma -1\right)}^{2}\right],\gamma <1.25\\ 1.6\gamma^{-0.4},\gamma \geq 1.25 \end{array}\right)$$

Where, $U_{\max}$ is the maximum velocity of the cavity jet, ρ is the density of the surrounding fluid, ΔP is the driving pressure, that is initial pressure inside the cavity and surrounding liquid and γ is the stand-off parameter.

### 3.4 Effect of size ratio (β)

**Fig. 12:**
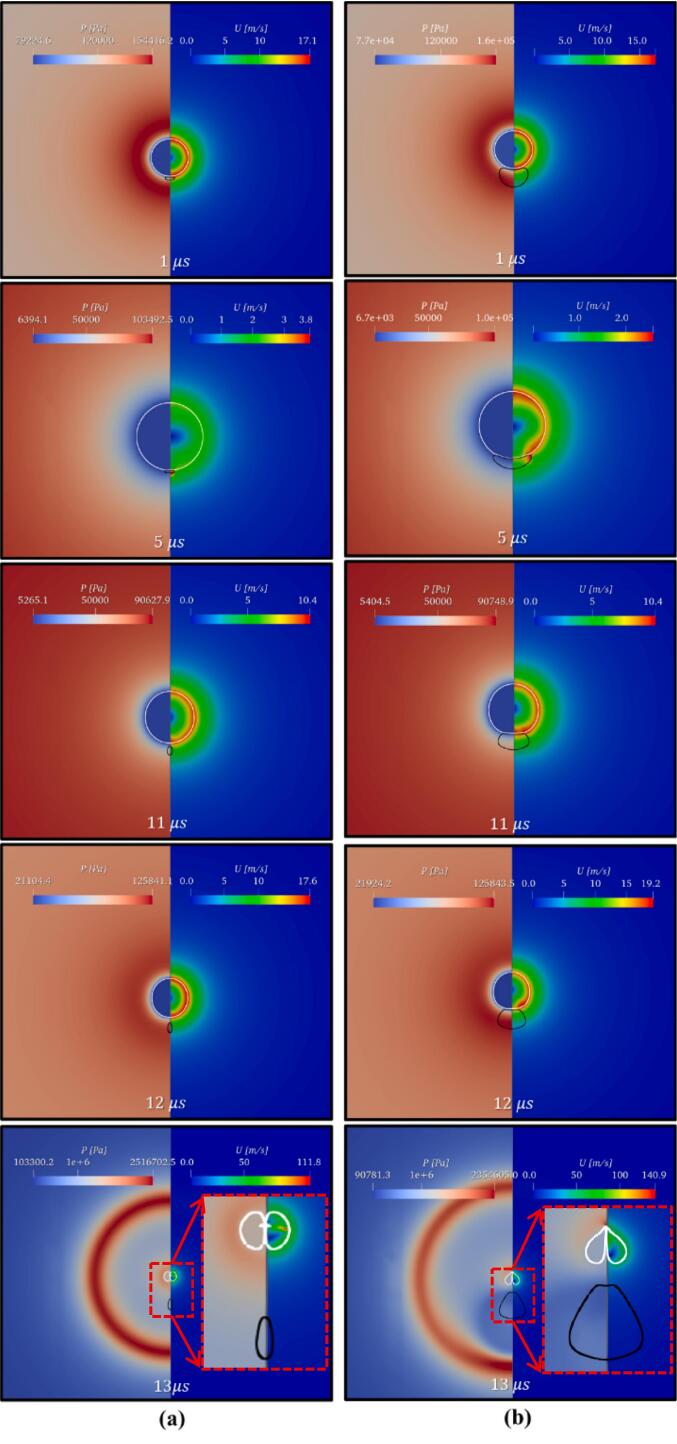
Pressure (Left) and velocity (Right) contour for different droplet-to-cavity size ratios (β) (a) β=0.625 (γ=2.6); (b) β=2.5 (γ=1.4).

**Fig. 13:**
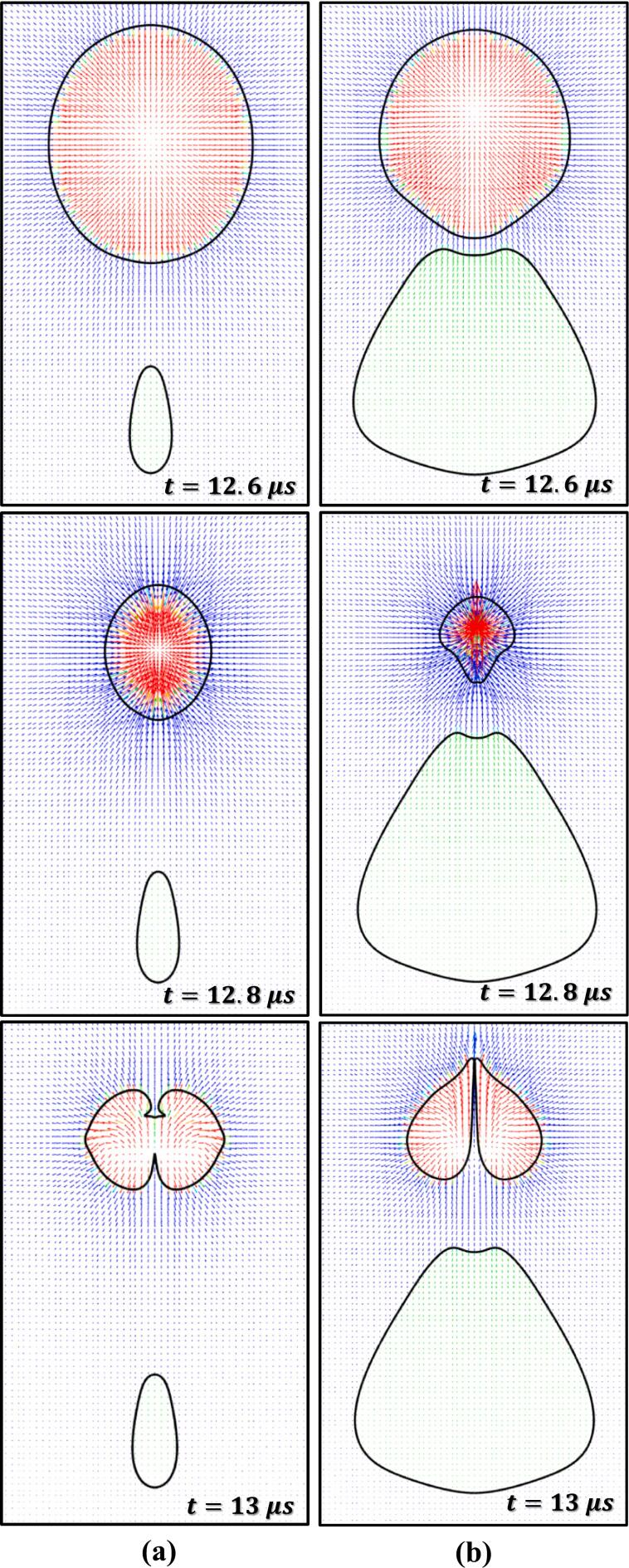
Velocity vector for β=0.625atγ=2.6 and β=2.5atγ=1.4 for t=12.6 μs, 12.8 μs and 13 μs.

This section discusses the effect of size ratio (β) on the flow field, cavity jet velocity, and droplet deformation. The size ratio is varied by changing $R_{d}$ in β and keeping L constant (10 μm). Since the cavity dynamics during the first expansion of the cavity remain similar to the case discussed in Section 3.2. Therefore, the cavity dynamics during the cavity collapse are discussed in this section. The pressure and velocity fields generated during the cavity droplet interaction for β=0.625 (γ=2.6) and 2.5 (γ=1.4) are shown in Fig. 12. Fig. 13 represents the velocity vector plot for the same cases (=0.625 and 2.5) but for time steps 12.6,12.8 and 13 µs, respectively.

In the case of β=0.625, the oil droplet size is very small as compared to the cavity, therefore during the cavity collapse there is no influence of the oil droplet on the cavity. In Fig. 12(a) (t=12 µs) and 13(a) (t=12.6 µs and 12.8 µs), it can be observed that the oil droplet elongates but does not disturb the flow field significantly. Moreover, the presence of the oil droplet does not affect the flow field in the immediate vicinity of the cavity. Therefore, the cavity collapses symmetrically, that is without cavity jet formation as shown in Fig. 12(a) and Fig. 13(a) at t=13 µs. When β=2.5, then during the cavity collapse, the pressure difference is generated across the cavity (as discussed in Section 3.2). This developed pressure difference across the cavity facilitates the formation of the cavity jet during the first collapse cycle (Fig. 12(a) at t=12 µs). The cavity jet creates a suction effect and pulls the surrounding fluid present in its immediate vicinity (refer to Fig. 12(b) at t=12.8 µs). Because of this pressure difference across the cavity, a flow merging effect is created. As discussed in the previous section, the flow merging effect is responsible for both the phenomena, that is cavity jet formation and droplet deformation. The magnitude of the cavity jet velocity is high (∼156 m/s).

**Fig. 14:**
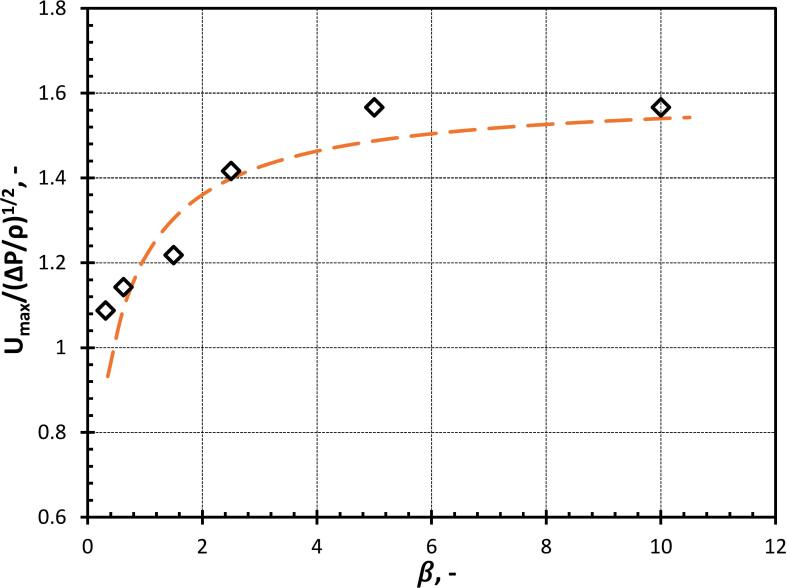
Normalized cavity jet velocity for different droplet-to-cavity size ratios (β).

The variation of the normalized maximum velocity with size ratio (β) is shown in Fig. 14. When β<1, the cavity collapses symmetrically because the influence of the oil droplet on the flow field in the immediate vicinity of the cavity is negligible as shown in Fig. 13(a). When β>1, the cavity collapses asymmetrically leading to a strong jet due to the influence of the oil droplet on the flow field of the cavity. This influence of the oil droplet in the flow field of the cavity is responsible for the generation of the pressure difference across the cavity which leads to the flow merging effect of the surrounding fluid as Fig. 13(b). The correlation for the change in the normalized maximum velocity with β is derived from Equation 16 and is given as:

(17) Umax/ΔPρ1/2=1.61+1/β-0.4

It can be seen that the cavity jet velocity does not change significantly with further increase in β beyond 2.5. The results shown in Fig. 14 show that the change in predicted jet velocity beyond β = 2.5 is less than 13 %. Considering this, it is reasonable to assume that cavity jet velocity is not sensitive to the shape of the boundary (planar or curvature) for β≥2.5.

### 3.5 Energy dissipation rate (ε)

Energy dissipation rate (ε) plays an important role in controlling the droplet size distribution in the liquid–liquid emulsification process. The ε is essential because it is related to the droplet breakage. The droplet breakage occurs via interactions with the eddies with a length scale smaller than or equal to the droplet size (d) and sufficient energy dissipation (ε) [49], [50], [51]. The droplet breakage caused via interaction with turbulent eddies will continue and eventually approach the minimum droplet size, $d_{\min}$. The minimum droplet size is related to the turbulent energy dissipation rate [50], [52], [53], [54]:

(18) $$d_{\min}\propto \varepsilon^{-0.4}$$

The drop breakage rate (k) is usually formulated as a complex function of energy dissipation rates and droplet diameter [49], [55]:

(19) $$k\;\alpha \;\frac{\varepsilon^{1/3}}{d^{2/3}}\;P\left(\varepsilon ,\;d\right)$$

Where P(ε,d) is a breakage probability.

The energy dissipation rates influence the drop breakage and thus influence the daughter size distribution of a single droplet. This in turn influences the entire droplet size distribution in the liquid–liquid emulsion. The relationship between ε and droplet size in the liquid–liquid emulsification process has been shown by Thaker and Ranade [7], for hydrodynamic cavitation-based fluidic devices. Thaker and Ranade [10] show the influence of the pressure drop and flow rate on the ε and droplet size. Moreover, Pandey et al. [56] have shown the influence of the ε on the breakage frequency/rate for different fluidic devices such as fluidic oscillators (low energy dissipation rate), helical coil (medium energy dissipation rate), and vortex diode (high energy dissipation rate). In the present work, the ε is obtained using the large velocity gradient generated inside the continuous medium because of the cavity collapse. The ε [57], [58] is calculated using:

(20) $$\varepsilon =\frac{1}{2}v\overset{¯}{{\left(\frac{\partial u_{i}}{\partial x_{j}}+\frac{\partial u_{j}}{\partial x_{i}}\right)}^{2}}$$

**Fig. 15:**
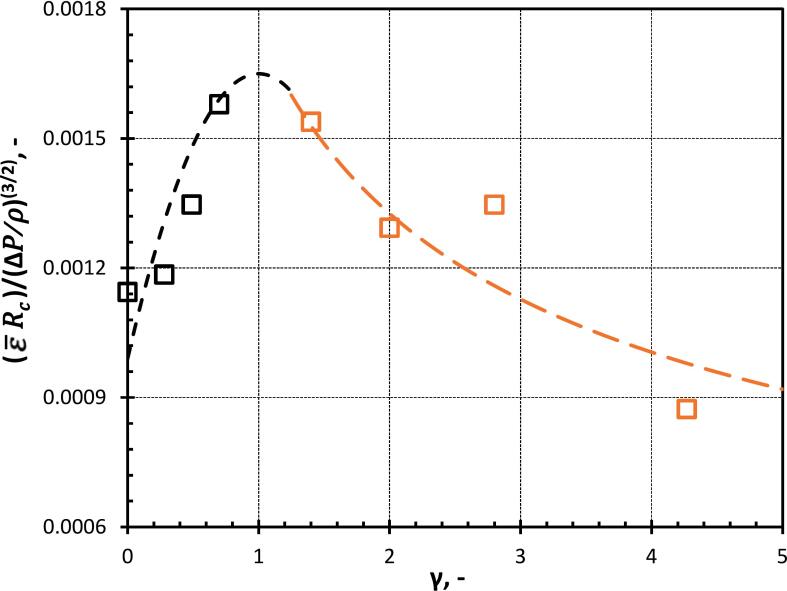
Variation of averaged energy dissipation rate with γ (Dashed lines represent Equations 21 (γ<1.25, Black colour and γ≥1.25, Orange colour), and the symbols denote the corresponding simulation result obtained using Equation 20).

The $\overset{¯}{\varepsilon }$ represents the volume-weighted average energy dissipation rate (refer to Equation 21). Fig. 15 shows the variation of $\overset{¯}{\varepsilon }$ with γ. $\overset{¯}{\varepsilon }$ increases till γ<1 (Equation 21) and thereafter decreases, this is because the cavity jet velocity increases due to flow merging which contributes to an increase in $\overset{¯}{\varepsilon }$. Thereafter, as the flow merging effect decreases because of an increase in the distance between cavity and droplet and also due to the large deformation of the oil droplet, the $\overset{¯}{\varepsilon }$ also starts to decrease. $\overset{¯}{\varepsilon }$ for all the cases is of the order $\;10^{8}$ m^2^/s^3^. A correlation has been proposed for $\overset{¯}{\varepsilon }$ and γ as:

(21) $$\frac{\overset{¯}{\varepsilon }\;R_{c}}{{\left(\Delta P/\rho \right)}^{3/2}}=\left\{\begin{array}{c} \left(1.65\;x\;10^{-3}\right)\left[1-0.4{\left(\gamma -1\right)}^{2}\right],\gamma <1.25\\ \left(1.75\;x\;10^{-3}\right)\gamma^{-0.4},\gamma \geq 1.25 \end{array}\right)$$

**Fig. 16:**
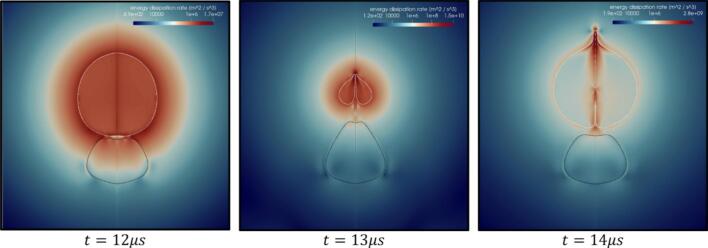
Contour of ε (β=2.5,γ=1.4) at t=12 μs, 13 μs and 14 μs.

Fig. 16 shows the change in the ε for β=2.5 at t=12 μs, 13 μs and 14 μs, i.e., before, during and after the formation of the cavity jet respectively. At t=12 μs, the cavity is in the compression stage, which is why the maximum ε is observed at the interface of the cavity-droplet. The relative movement between the cavity and the continuous medium leads to significant viscous force. This viscous force will be highest near the tip of the cavity jet and thus maximum ε is generated at the cavity jet tip (Fig. 16, t=13 μs). At t=14 μs, even though the cavity has expanded the flow of the continuous medium through the cavity is taking place which is why ε can still be observed near the cavity tip region.

## 4 Conclusions

Present study investigates the cavity dynamics near a deformable oil droplet using the multi-fluid VOF method. Axisymmetric simulations were performed in a cylindrical domain where dispersed phases, cavity, and oil droplet are initially placed in a continuous medium (water). The cavity oscillations were initiated by specifying high pressure in the cavity as an initial condition ($10^{7}$ Pa) and atmospheric conditions everywhere else. The primary objectives are to understand the physics behind the cavity-droplet interaction and quantify the influences of stand-off parameter (γ) and size ratios (β) on the cavity dynamics and droplet deformation. An attempt has also been made to quantify the average energy dissipation rates $\left(\overset{¯}{\varepsilon }\;\right)$ generated by single cavity collapse. The following conclusions have been drawn:

• In this work, the direction of the cavity jet is always towards the denser medium, that is, water.
• The velocity magnitude of the cavity jet depends on the size ratio (β) and stand-off parameter (γ) as these two factors affect the flow merging phenomenon.
• A non-monotonic behaviour has been observed for the normalized cavity jet velocity with γ. The normalized cavity jet velocity increases till γ=1 and thereafter decreases. A correlation has been proposed for this behaviour.
• No cavity jet formation occurs when β<1, thereafter with the increase in β, the normalized cavity jet velocity also increases. The change in the normalized cavity jet velocity with β follows the power law.
• The average energy dissipation rate generated during the cavity collapse is of the order $\;10^{8}$ m^2^/s^3^.

The presented approach and results provide new insight into the cavity-droplet interactions, which will be useful in understanding droplet deformation leading to its breakage and eventually the formation of the emulsions using hydrodynamic cavitation-based fluidic devices.

## CRediT authorship contribution statement

**Deepak K. Pandey:** Writing – original draft, Visualization, Validation, Methodology, Investigation, Formal analysis, Data curation. **Rupak Kumar:** Writing – original draft, Visualization, Validation, Methodology, Investigation, Formal analysis, Data curation. **Vivek V. Ranade:** Writing – review & editing, Supervision, Project administration, Funding acquisition, Conceptualization.

## Declaration of competing interest

The authors declare that they have no known competing financial interests or personal relationships that could have appeared to influence the work reported in this paper.

## Data Availability

The data supporting this study’s findings are available from the corresponding author upon reasonable request.
