# Supplementary material for: Understanding cavity dynamics near deformable oil drop via numerical simulations
Source: Ultrason Sonochem. 2025 Mar 24;116:107325. doi: 10.1016/j.ultsonch.2025.107325 (PMC11987696; doi:10.1016/j.ultsonch.2025.107325)
Supplement: Supplementary Data 1 [file mmc1.pdf]

Supplementary Information

Understanding cavity dynamics near deformable oil drop via numerical simulations

Deepak K. Pandey+, Rupak Kumar+ and Vivek V. Ranade*
Multiphase Reactors and Intensification Group
Bernal Institute, University of Limerick, Limerick V94T9PX, Ireland
+ Equal contributions; *Email: vivek.ranade@ul.ie

S1. Details for simulation of cavity-droplet interaction in OpenFOAM

Table S1: Setting fvSolution

No.	Parameter			
1	alpha.*	nAlphaSubCycles		4
		cAlpha		1
		limitAlpha		0.99
		solver		smoothSolver
		smoother		GaussSeidel
		tolerance		1e-8
		relTol		0
2	pcorr.*	solver		PCG
		preconditioner		
			preconditioner	GAMG
			tolerance	1e-5
			relTol	0
			smoother	GaussSeidel
		tolerance		1e-5
		relTol		0
		maxIter		100
3	.*(rho|rhoFinal)	solver		diagonal
4	p_rgh	solver		GAMG
		smoother		1e-5
		tolerance		0.05
		relTol		GaussSeidel
5	p_rghFinal	solver		PCG
		preconditioner		
			preconditioner	GAMG
			Tolerance	1e-7
			relTol	0
			nVcycles	2
			Smoother	GaussSeidel
		tolerance		1e-7
		relTol		0
		maxItr		20

6	(U|T|k|B|nuTilda)	solver		smoothSolver
		smoother		symGaussSeidel
		tolerance		1e-8
		relTol		0.1
		nSweeps		1
7	(U|T|k|B|nuTilda)Final	$U		0
		relTol		0
8	PIMPLE	nCorrectors		2
		nNonOrthogonalCorrectors		0
9	relaxationFactors	equations		
			U.*	1

Table S2: Setting fvSchemes

S. No.	Parameter		
1	dtSchemes	default	Euler
2	gradSchemes	default	pointCellsLeastSquares
3	divSchemes		
		div(rhoPhi, U)	Gauss upwind
		div(phi, alpha)	Gauss vanLeer
		div(phirb, alpha)	Gauss linear
		div(rhoPhi, T)	Gauss upwind
		div(rhoPhi,K)	Gauss upwind
		div(phi, p)	Gauss upwind
		div(((rho*nuEff)*dev2(T(grad(U)))))	Gauss linear
4	laplacianSchemes	default	Gauss linear corrected
5	interpolationSchemes	default	linear
6	snGradSchemes	default	corrected

S2. Post-processing

The post-processing of the simulation results is an important aspect. To obtain the velocity field, pressure fields, and energy dissipation rate from the DNS of a single cavity-oil droplet, the following procedure was followed.

- The volume fraction of the cavity and droplet, velocity field, pressure field, and other scalar quantity files were stored for each time step during the simulation.
- These files were exported to ParaView for visualization, analysis, and calculation of dependent quantities such as energy dissipation rate (ε).

- The cell-centered data of volume fraction, pressure, and velocity were extracted, and their respective contours were plotted for each time step.
- The cell-centered value of maximum velocity in the domain and volume fraction of the oil droplet was calculated for each time step using the filter ‘PlotDataOverTime.’
- The ‘ComputeDerivatives’ filter was used to calculate the spatial derivatives of the velocity components.
- The energy dissipation rate was computed using the ‘Calculator’ filter.
- Maximum energy dissipation rate at each time step was obtained utilizing the ‘PlotDataOverTime’ filter.
- All the above steps were repeated for different size ratios and stand-off parameters.
